# Antimalarial Activity of Methanolic Leaf Extract of *Piper betle* L.

**DOI:** 10.3390/molecules16010107

**Published:** 2010-12-28

**Authors:** Abdulelah H. Al-Adhroey, Zurainee M. Nor, Hesham M. Al-Mekhlafi, Adel A. Amran, Rohela Mahmud

**Affiliations:** 1 Department of Parasitology, Faculty of Medicine, University of Malaya, Kuala Lumpur 50603, Malaysia; E-Mails: zuraineemn@um.edu.my (Z.M.N.); halmekhlafi@yahoo.my (H.M.A.); rohela@ummc.edu.my (R.M.); 2 Department of Physiology, Faculty of Medicine, Universiti Kebangsaan Malaysia, Jalan Raja Muda Abdul Aziz, Kuala Lumpur 50300, Malaysia; E-Mail adel.emran@yahoo.com (A.A.A.)

**Keywords:** *Piper betle*, methanolic extract, antimalarial activity

## Abstract

The need for new compounds active against malaria parasites is made more urgent by the rapid spread of drug-resistance to available antimalarial drugs. The crude methanol extract of *Piper betle* leaves (50–400 mg/kg) was investigated for its antimalarial activity against *Plasmodium berghei*(NK65) during early and established infections. The phytochemical and antioxidant potentials of the crude extract were evaluated to elucidate the possibilities of its antimalarial effects. The safety of the extract was also investigated in ICR mice of both sexes by the acute oral toxicity limit test. The leaf extract demonstrated significant (*P* < 0.05) schizonticidal activity in all three antimalarial evaluation models. Phytochemical screening showed that the leaf extract contains some vital antiplasmodial chemical constituents. The extract also exhibited a potent ability to scavenge the free radicals. The results of acute toxicity showed that the methanol extract of *Piper betle* leaves is toxicologically safe by oral administration. The results suggest that the Malaysian folklorical medicinal application of the extract of *Piper betle* leaf has a pharmacological basis.

## 1. Introduction

Malaria, a tropical blood-borne protozoan disease caused by parasites of the genus *Plasmodium,* is one of the most important infectious diseases in the World. Nowadays, anti-malarial drug resistance has become one of the most important challenges to malaria control efforts [1]. Drug resistance is responsible for the spread of malaria to new areas, the recurrence of malaria in areas where the disease had been eradicated and plays an important role in the occurrence and severity of epidemics in some parts of the World [2]. Considering this growing problem, there is a broad consensus as to the need to develop new anti-malarial drugs which can cope with the spread of drug resistant malarial parasites [3,4]. The ethnomedical approach to the search for new anti-malarial drugs from plant sources has proved to be more predictive, where the most important modern anti-malarial drugs are derived from the medicinal plants known to have ethnomedical standing [5,6,7,8]. 

*Piper betle* Linn is a liana belonging to the Piperaceae family. It is cultivated in most of South and Southeast Asia and valued both as a mild stimulant and for its medicinal properties. In Peninsular Malaysia, the leaves of *Piper betle* are also used by the rural population as an antimalarial remedy [9]. The leaves are chewed alone or with other plant materials. Recently, the leaves have been reported to exhibit many pharmacological effects including anti-bacterial [10], anti-leishmanial [11], anti-filarial [12] and anti-fungal [13] properties. This study, therefore, aims to find out if *Piper betle* leaves possess *in vivo* anti-malarial effects against the laboratory malaria model, *Plasmodium berghei*.

## 2. Results and Discussion

### 2.1. Phytochemical screening

Phytochemical screening of the methanol extract of *Piper betle* leaves revealed that the leaf extract contains alkaloids, terpenes, anthraquinones, flavonoids, tannins, saponins and steroids. Phytochemical compounds such as alkaloids are commonly implicated in the antiplasmodial activity of many plants [14,15,16,17]. Terpenes or terpenoids have been identified as active antiprotozal and antimalarial agents in many pharmacological studies [18,19,20,21]. Flavonoids are the other forms of *Piper betle* phenolic structures. Flavonoids revealed significant anti-parasitic activities against different parasite strains of malaria, trypanosome and leishmania [22,23,24]. Derivatives of 9,10-anthraquinone include many important drugs including antimalarials like rufigallol [25]. These chemical compounds which were found in this extract may be acting singly or in synergy with one another to exert the observed antiplasmodial activity of *Piper betle*. 

### 2.2. Antioxidant capacity

**Figure 1:**
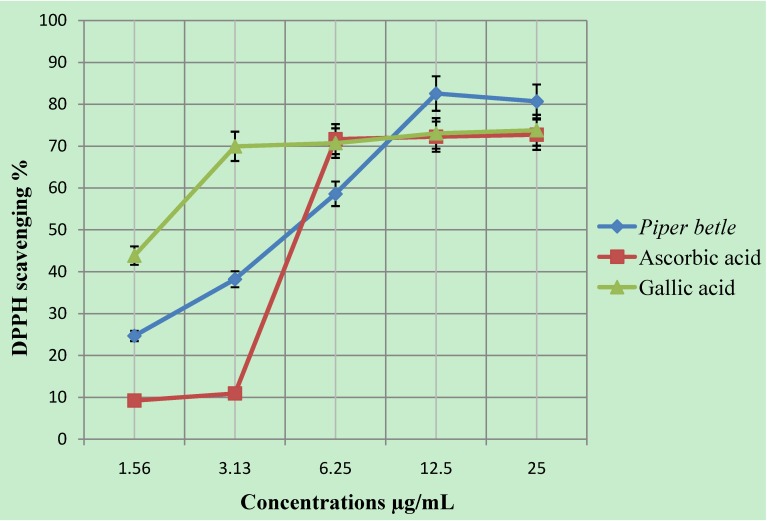
DPPH radical scavenging activity of methanol extract of *Piper betle* leaves. Absorbance values represent means of triplicate tests of the different concentrations analyzed.

Investigation on the antioxidant activity of the extract against 1,1-diphenyl-2-picrylhydrazyl radicals (DPPH) showed that it exhibited a strong radical scavenging capacity, comparable to that of controls. At a concentration of 12.5 µg/mL, the scavenging activity of the methanol extract of the leaves reached 82.56 ± 1.50 %, while at the same concentration, those of the ascorbic and gallic acids controls were 72.25 ± 2.44 and 73.03 ± 2.27 %, respectively (Figure 1). The potent antioxidant capacity exhibited by the *Piper betle* leaves may be due to the phenolic compounds in this extract, such as chavicol, chavibetol, chavibetol acetate and eugenol [26,27,28]. The antioxidant effect of the *Piper betle* leaf extract may represent another mechanism that contributes to its anti-malarial activity. *Piper betle* leaf extract decreases nitric oxide (NO) production in macrophages [29]. According to Daubener [30], the inhibition of NO starves the parasite of an essential amino acid, leading to its death by increasing tryptophan degradation through indolamine deoxygenase induction in human peritoneal macrophages. 

### 2.3. Median lethal dose

**Figure 2:**
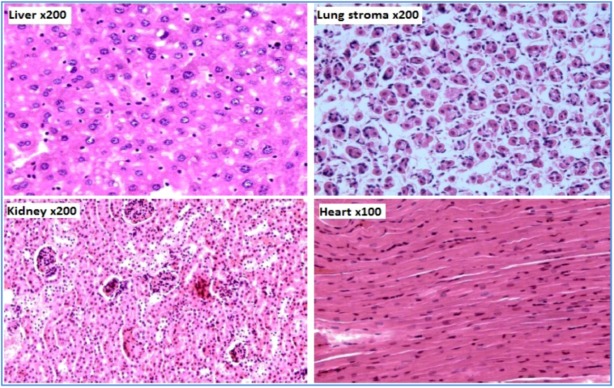
Photomicrographs of the sections of the liver × 200, lung stroma × 200, kidney × 200 and heart × 100 showing normal features in mice treated with 5,000 mg/kg of methanol extract of *Piper betle* leaves.

No deaths occurred during the observation period. Some symptoms were observed 1 hour following administration of the extract. Ataxia and piloerection were noted in males and females; all of which resolved by 3 hours after administration. No abnormal general symptoms were observed in the extract treated groups. All animals gained body weight at the end of the 14-day observation period. Administration of the leaf extract at 5,000 mg/kg had no effect on the necropsy findings. Histopathological lesions were not observed in any of the main organs of the mice (Figure 2). 

Based on these results the median lethal dose (LD_50_) of the methanol extract of *Piper betle* leaf after single oral administration in male and female ICR mice was found to be greater than 5,000 mg/kg body weight. The LD_50_ value of more than 5,000 mg/kg showed that the extract is practically safe [31,32].

### 2.4. In vivo antimalarial activity

The leaf extract demonstrated significant (*P* < 0.05) schizonticidal activity in the all three models of the antimalarial evaluations. The suppression of parasitaemia by chloroquine at 20 mg/kg/day was in agreement with a previous study by Muregi *et al*. [33].

#### 2.4.1. Suppressive anti-malarial activity

The *in vivo* antiplasmodial activity of four different doses (50, 100, 200, and 400 mg/kg) of the methanol extract of *Piper betle* leaves administrated orally showed a dose-dependent chemosuppressive activity which ranged between 36.47% to 82.31 % in all groups of mice (Table 1). A considerably high degree of chemosuppression was shown by the 200 and 400 mg/kg doses which significantly decreased the parasitaemia of the infected mice when compared to control (*P* < 0.01).

**Table 1:** Effects of methanol extract of *Piper betle* on early malaria infection.

Extract/drug	Dose	% Parasitaemia	%Chemo-suppression	Significance
Control (dist. water)	0.2 mL	5.10 ± 0.33		
*Piper betle Leaf* extract	50 mg/kg	3.24 ± 0.82	36.47	ns
	100 mg/kg	2.40 ± 0.68	52.94	*P* < 0.05
	200 mg/kg	1.50 ± 0.63	70.51	*P* < 0.01
	400 mg/kg	0.90 ± 0.33	82.31	*P* < 0.01
Chloroquine	20 mg/kg	0.00	100	

#### 2.4.2. Curative anti-malarial activity

**Figure 3:**
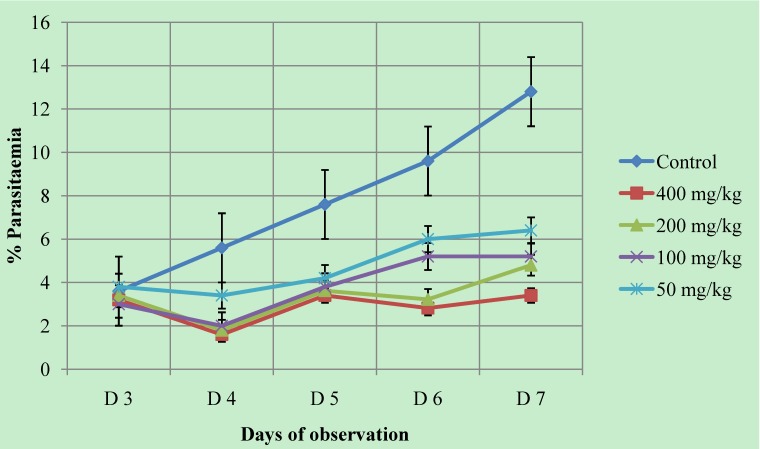
Comparison of parasitaemia chemosuppression of the extract-treated groups and control from D3 until D7 after infection.

The results indicated that the methanol extract of *Piper betle* leaves exhibited dose-dependent chemosuppression in parasitaemia. This curative chemosuppression of the treated groups was statistically significant (*P* < 0.05) when compared to the control. The control group showed daily increases in parasitaemia reaching 12.8 % on the seventh day of infection (Figure 3). The chemosuppression effects for the treated groups on the sixth day of infection were 37.50, 45.83, 66.46 and 70.63 for 50, 100, 200 and 400 mg/kg, respectively (Table 2). These treated groups (50 to 400 mg/kg) of mice also had longer survival times which ranged between 14.40 ± 0.93 days and 19.00 ± 1.22 days as compared to the control with 13.6 ± 0.82 days. The chloroquine-treated group had a mean survival time of 27.20 ± 2.33 days (Table 3).

**Table 2:** Activity of *Piper betle* on established malaria infection.

Extract/drug	Dose	% Parasitaemia	%Chemo-suppression	Significance
Control (dist. water)	0.2 mL	9.60 ± 0.93		
*Piper betle Leaf* extract	50 mg/kg	6.00 ± 0.84	37.50	*P* < 0.05
	100 mg/kg	5.20 ± 0.92	45.83	*P* < 0.05
	200 mg/kg	3.22 ± 0.95	66.46	*P* < 0.001
	400 mg/kg	2.82 ± 0.84	70.63	*P* < 0.05
Chloroquine	20 mg/kg	00	100	

**Table 3:** Mean survival period of mice treated with *Piper betle* in established malaria infection.

Drug/extract	Dose	Survival time (days)	Significance
Control (dist. water)	0.2 mL	13.60 ± 0.51	
*Piper betle Leaf* extract	50 mg/kg	14.40 ± 0.93	ns
	100 mg/kg	15.20 ± 1.16	ns
	200 mg/kg	17.20 ± 1.56	ns
	400 mg/kg	19.00 ± 1.22	*P* < 0.01
Chloroquine	20 mg/kg	27.20 ± 2.33	*P* < 0.001

#### 2.4.3. Prophylactic anti-malarial activity

The methanol extract of *Piper betle* exerted a dose-dependent prophylactic activity at the different doses employed resulting in significant (P < 0.05) reduction of parasitaemia in extract treated groups when compared to control. It exerted 19.57, 34.78, 52.17 and 70.88% chemosuppressions at doses of 50, 100, 200 and 400 mg/kg, respectively. The chemosuppression shown by the highest dose of the extract (400 mg/kg) was comparable to that of the standard drug, pyrimethamine with chemosuppression of 73.04% (Table 4).

**Table 4:** Effect of the methanol extract of *Piper betle* on residual malaria infection.

Drug/extract	Dose	% Parasitaemia	%Chemo-suppression	Significance
Control (dist. water)	0.2 mL	4.60 ± 0.51		
*Piper betle Leaf* extract	50 mg/kg	3.70 ± 0.30	19.57	ns
	100 mg/kg	3.00 ± 1.34	34.78	ns
	200 mg/kg	2.20 ± 0.37	52.17	*P* < 0.05
	400 mg/kg	1.34 ± 0.41	70.88	*P* < 0.001
Pyrimethamine	1.2 mg/kg	1.24 ± 0.47	73.04	*P* < 0.05

## 3. Experimental

### 3.1. Plant materials

The plant part (leaves) of *Piper betle* was selected based on an ethnobotanical survey conducted by the Department of Parasitology, Faculty of Medicine, University of Malaya, in two malaria endemic communities, forest-aboriginal and rural communities, in the Lipis district of Pahang state, Peninsular Malaysia [9]. The plant has been recorded as a curative and prophylactic antimalarial remedy by the two communities. The voucher specimen was collected and identified by the University of Malaya Herbarium (KLU) and deposited under the reference number KLU 046620.

### 3.2. Preparation of plant extract

The leaves of *Piper betle* were dried in a hot air oven at 40 °C and milled. Five hundred grams of the powdered leaves was soaked with absolute methanol (3.5 L, Merck, Germany) for 72 h. The extracts were concentrated in *vacuo* to dryness at 40 °C using a rotary evaporator. The percentage yield was 8.69%. The dry extract was stored in a refrigerator at 4 °C until used.

### 3.3. Animals

The animals (ICR mice), both male (27 ± 2 g) and female (22 ± 2 g), 6–7 weeks old which were used for these experiments were obtained from the Laboratory Animal Centre of the Faculty of Medicine, University of Malaya. The mice were housed in standard conditions and were maintained on a standard pelleted feed and water *ad libitum*. Permission and approval for animal studies were obtained from the Animal Ethics Committee of the Faculty of Medicine, University of Malaya dated 05 June 2009 (Ref. No. PAR/05/6/2009/AHAA-R).

### 3.4. Phytochemical screening

The chemical constituents of the leaf extract were tested for the presence of alkaloids, anthraquinones, terpenoids, flavonoids, tannins, steroids, saponins and glycosides using Dragendorff’s reagent, sulphuric acid-chloroform layer test, Salkowski’s test, ammonium test, lead sub-acetate reagent, Salkowski’s test, frothing test and Fehling’s reagent standard procedures, respectively [34,35]. 

### 3.5. DPPH radical scavenging activity

The antioxidant capacity of the extract was determined photometrically through its scavenging activity against the stable artificial free radical 1,1-diphenyl-2-picrylhydrazyl (DPPH), using the method of Gerhauser *et al*. [36]. In brief, DPPH (195 μM) was added to different concentrations of the extract (5 μL) in a 96-well microplate, and incubated for 3 hours and read at 20-min intervals. The bleaching of DPPH was observed at an absorbance of 515 nm. Ascorbic acid and gallic acid were used for comparison. The free radical-scavenging activity was expressed as the percentage of scavenging of the DPPH by the extract and was calculated as follows:

DPPH radical scavenging activity (%) = {[Ab-Aa]/Ab} × 100

where Ab is the absorption of the blank sample, and Aa is the absorption of the sample. Each test was carried out three times in quadruplicate and the mean was calculated. The DPPH % was presented as μg /mL of concentration.

### 3.6. Median lethal dose

Acute oral toxicity of the methanol extract of *Piper betle* leaves was studied according to the OECD guideline No 423 “Acute oral toxicity – acute toxic class method” [37]. A limit test was performed; one female and one male ICR mouse was administrated at 5,000 mg/kg. Since these mice survived, four additional mice were sequentially dosed at approximately 48 to 72-hour intervals. A total of five female and five male mice were tested. The mice were fasted overnight prior to administration and returned to feeding 3 hours later. On the day of extract administration, all the mice were observed for mortality and signs of toxicity at 1, 3 and 4 hours following administration and thereafter they were observed twice a day for 14 days. Body weights of the mice were recorded on study days -1, 0 (initiation), 7 and 14 (termination). All experimental mice were sacrificed at the end of the observation period and subjected to complete gross necropsy and histopathological study.

### 3.7. Parasite inoculation

Donor mouse blood infected with the *P. berghei* was used for inoculum preparation. The desired blood volume was drawn from the donor mouse by heart puncture and diluted serially in Alsever’s solution. The final suspension would contain about 1 × 10^6^ infected RBC’s in every 0.2 mL suspension. This 0.2 mL suspension was injected into mice intraperitoneally to initiate infection [38]. The inoculated animals were then randomized into five mice per cage and maintained in the Animal Room, Department of Parasitology, Faculty of Medicine, University of Malaya, in accordance with the internationally accepted principles for laboratory animals use and care.

### 3.8. In vivo antimalarial assays

A series of *in vivo* antimalarial assays were carried out to evaluate the *in vivo* anti-malarial activities of the methanolic extract of *Piper betle* leaves at 50, 100, 200 and 400 mg/kg doses as compared to control groups treated with distilled water (containing 10% DMSO, the solvent of the test extracts) and reference groups treated with standard drugs (chloroquine 20 mg/kg or pyrimethamine 1.2 mg/kg). Malaria infection was first established in female mice by the intraperitoneal (i.p.) administration of donor female ICR mouse blood containing about 1 × 10^6^ parasites. The percentage parasitaemia was determined by counting the parasitized red blood cells out of 9000 RBC’s in random fields of the microscope:

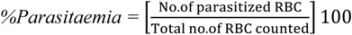



Average percentage chemosuppression was calculated as:

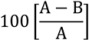

where A is the mean percentage parasitaemia in the negative control group and B is the mean percentage parasitaemia in the test group.

#### 3.8.1. 4-day suppressive activity (early malaria infection)

Suppressive activity of the extract was assessed using the method described by Peters and Robinson [39]. Thirty female ICR mice were first inoculated intraperitoneally with 0.2 mL suspension containing 1 × 10^6^ *P. berghei* on the first day (D0). After 2–4 hours post-infection, the experimental groups were treated orally with 50, 100, 200 and 400 mg/kg/day doses of the extract. The reference drug group was treated with chloroquine (20 mg/kg) and the control group received distilled water 0.2 mL/kg. All the treatments were repeated for the next three days (D1 to D3). On the fifth day (D4), thin blood smears were prepared from each mouse and stained with Giemsa’s stain.

#### 3.8.2. Curative activity (established malaria infection)

Thirty mice were selected and inoculum of 0.2 mL (1 × 10^6^ *P. berghei*) was given to each mouse (i.p.). The mice were then regrouped into six groups of five mice each. Seventy-two hours later, the experimental groups were treated orally with 50, 100, 200 and 400 mg/kg/day doses of the extract. The reference drug group was treated with chloroquine (20 mg/kg) and the control group received distilled water 0.2 mL. The treatments were continued daily until D7. Thin blood smears were collected daily from tail blood, stained with Giemsa’s stain and examined microscopically for determination of parasitaemia. On the sixth day of the infection, the mean percentage suppression of parasitaemia was calculated according to the procedure described by Ryley and Peters [40] and Saidu *et al*. [41]. The mean survival time (days) for each group was determined over a period of 30 days post-infection.

#### 3.8.3. Prophylactic activity (residual malaria infection)

The prophylactic activity of the extracts was assessed using the method described by Peters [42]. The experimental mice were randomly divided into six groups of five mice each. The mice were administered orally with 50, 100, 200 and 400 mg/kg/day of the extract, pyrimethamine 1.2 mg/kg/day was administered to the reference drug group, and distilled water 0.2 mL to the control group. The treatment was given for 3 consecutive days (D0–D2). On the fourth day (D3), all mice were infected with 1 × 10^6^ *P. berghei* and kept for the next 3 days. On D7, blood smears were prepared from the tail blood. The percentage of suppression of parasitaemia was then calculated.

### 3.9. Statistical analysis

All data were expressed as mean ± S.D. and S.E.M. of triplicate parallel measurements. Statistical analysis was performed using Students’ t-test and ANOVA (one- or two-way). Differences between means at 1 and 5% level (*P* ≤ 0.01 and 0.05) were considered significant.

## 4. Conclusions

The leaf extract of *Piper betle* demonstrated significant (*P* < 0.05) schizonticidal activity in all the three models of the antimalarial evaluations. The results of this study provide a basis for further studies on the plant. These include the isolation and characterization of the bioactive principles with the ultimate objective of finding novel antimalarial compounds which can be used in the fight against drug resistant malaria. This study also suggests that the Malaysian folkloric medicinal application of the extract of *Piper betle* leaves has a pharmacological basis.
